# COMPARATIVE ANALYSIS OF OPEN AND CLOSED FLOATING KNEE INJURIES

**DOI:** 10.1590/1413-785220233104e262810

**Published:** 2023-07-31

**Authors:** BAHRI BOZGEYIK, ORHAN BÜYÜKBEBECI, SAVAŞ GÜNER, AHMET MERT

**Affiliations:** 1 Kadirli State Hospital, Department of Orthopedic Surgery, Osmaniye, Türkiye.; 2 Gaziantep University Hospital, Department of Orthopedic Surgery, Gaziantep, Türkiye.; 3 Ömer Halis Demir Unıversity Hospital, Department of Orthopedic Surgery, Niğde, Türkiye.

**Keywords:** Knee Injuries, Femur, Tibia, Fractures, Bone, Traumatismos do Joelho, Fêmur, Tíbia, Fraturas Ósseas

## Abstract

**Objective:**

To compare the functional outcomes between floating knee injuries with open femur and tibia fractures and closed floating knee injuries.

**Methods:**

Floating knee injuries (followed up and treated in our clinic) were retrospectively analyzed. Patients were divided into two groups: floating knee injuries with open femur and tibia fractures (Group 1) and floating knee injuries with closed femur and tibia fractures (Group 2). Patients were compared according to their demographic characteristics and clinical and functional outcomes.

**Results:**

Of 52 study patients, 28 had Group 1 injuries and 24, Group 2 injuries. We found a statistically significant difference in length of hospital stay between the two groups (p **=** 0.01) and a statistically significant difference in Karlström-Olerud functional scores between the groups (p **=** 0.02). We found osteomyelitis in five (17%) patients in Group 1 and in one (4%) patient in Group 2.

**Conclusion:**

Patients with floating knee injuries and open fractures showed poorer outcomes than those with closed fractures. Those with open floating knee injuries show complications more often and longer hospital stays. ***Level of Evidence III, Therapeutic Studies Investigating the Results of Treatment.***

## RESUMO

**Objetivo:**

Comparar os resultados funcionais entre lesões do tipo joelho flutuante com fraturas expostas de fêmur e tíbia e lesões de joelho flutuante fechadas.

**Métodos:**

As lesões de joelho flutuante acompanhadas e tratadas em nossa clínica foram analisadas retrospectivamente. Os pacientes foram divididos em dois grupos: lesões de joelho flutuante com fraturas expostas de fêmur e tíbia (Grupo 1) e lesões de joelho flutuante com fraturas fechadas de fêmur e tíbia (Grupo 2). Os pacientes foram comparados de acordo com as características demográficas e os desfechos clínicos e funcionais.

**Resultados:**

Entre os 52 pacientes do estudo, 28 tiveram lesões do Grupo 1 e 24 do Grupo 2. A diferença no tempo de internação entre os dois grupos foi estatisticamente significativa (p = 0,01). Também houve diferença estatisticamente significativa nos escores funcionais de Karlström e Olerud entre os grupos (p = 0,02). Osteomielite foi identificada em 5 (17%) pacientes do Grupo 1 e em 1 (4%) paciente do Grupo 2.

**Conclusão:**

Comparados aos pacientes com lesões de joelho flutuante com fraturas fechadas, aqueles com fraturas expostas têm piores resultados, uma vez que as complicações são mais comuns e a permanência hospitalar é mais longa nestes casos. **Nível de Evidência III, Estudos Terapêuticos - Investigação dos Resultados do Tratamento.**

## INTRODUCTION

The term floating knee, first described by Blake and McBryde,^1^ includes traumatic ipsilateral fractures of the femur and tibia. These injuries result from high-energy traumas and are usually associated with high rates of mortality and morbidity. ^(^ ^2^ ^),(^ ^3^ Fraser classified floating knee injuries in 1978 to guide their treatment. ^(^ ^4^ This classification sorts fractures based on their location in patients’ femur and tibia. Since floating knee injuries are high-energy injuries, patients may have additional injuries, which may include additional problems such as abdominal and thoracic injuries. ^(^ ^5^ Vascular injuries may also accompany these traumas, showing a rate of around 7%.^6^

The formation of fractures by high-energy mechanisms also damages the soft tissues surrounding the fractures. Therefore, many patients show open fractures. Treatment of patients with open injuries can be more complicated. A literature review shows several studies on the outcomes of floating knee injuries. ^(^ ^7^ ^),(^ ^8^ However, no study has evaluated both femoral and tibial fractures due to open injuries and compared open fractures with isolated closed ones.

This study aimed to compare the functional outcomes between adult-type open floating knee injuries and closed floating knee injuries.

## METHODS

Following the approval of the local ethics committee (numbered 2021/220), 52 patients with floating knee injury from 2013 to 2019 were retrospectively reviewed and included in this study. Among 52 study patients, 28 had open floating knee injuries (Group 1) and 24 (Group 2), closed fractures. Patients with open injuries were categorized by the Gustilo-Anderson classification. Fraser’s classification was used to classify both groups. Patients’ age, gender, neurovascular damage, follow-up length, union presence, fixation method, osteomyelitis development, hospital stay length, type of fracture fixation, and complications were recorded. All patients were evaluated by X-ray at follow-ups after 3, 6, 9, and 12 months. Functional outcomes in both groups were assessed by the Karlström-Olerud criteria. Children; pregnant women; patients with pathological fractures and isolated open femur or tibia fracture, and those who missed regular follow-ups were excluded. Adults with closed femur and tibia fractures (Group 2) and open femur and tibia fractures (Group 1) were included in this study. Patients with open fractures were administered first-generation cephalosporin and metronidazole during their hospital stay. Antibiotics were changed according to the culture results in eligible patients. The closed fracture group was preoperatively administered prophylactic first-generation cephalosporin.

### Statistical analysis

The descriptive statistics of the analyzed variables in this study were expressed as mean ± standard deviation and median (minimum-maximum) and nominal variables as n (%) in appropriate charts. The statistical significance of nominal variables between groups was tested using the chi-squared test and that of continuous variables, by the Mann-Whitney U test. In all statistical analyses, the level of significance was set at p < 0.05. IBM SPSS, version 22.0, (IBM Corp, Armonk, NY, USA) was used for data analysis.

## RESULTS

This study included 52 patients with floating knee injuries, 28 of which had open floating knee injuries and 24, closed fractures. Group 1 had 26 (92%) men and 2 (2%) women, whereas Group 2, 20 (83%) men and 4 (17%) women. Group 1 and 2 showed a 33.96 (18-59) and 32.7 (16-68) mean age (in years), respectively.

According to Fraser’s classification, 15 (28%) patients had Type I fractures; 12 (23%), Type IIa; 14 (27%), Type IIb; and 11 (21%), Type IIc. According to the Gustilo-Anderson classification, three patients in the open floating knee injury group had Type I fractures; seven, Type II; and 18, Type III femoral fractures (Table 1).

**Table 1:** Fraser and Gustilo-Anderson classifications by Fraser subtypes.

		Fraser Classification			
		Type I (n = 15)	Type IIa (n = 12)	Type IIb (n = 14)	Type IIc (n = 11)
Femur	Closed	7	6	6	5
Open fracture Gustilo-Anderson classification	Type I	2	0	0	1
	Type II	3	0	3	1
	Type IIIa	1	3	4	3
	Type IIIb	1	2	0	0
	Type IIIc	1	1	1	1
Tibia	Closed	7	6	6	5
Open fracture Gustilo-Anderson classification	Type I	0	0	0	0
	Type II	2	1	2	2
	Type IIIa	1	2	2	3
	Type IIIb	2	2	2	0
	Type IIIc	3	1	2	1
Age		28.06 (16-49)	33.08 (18-47)	35.07 (21-65)	38.81 (18-68)
Length of hospital stay (days)		7 (5-11)	12 (9-15)	13.71 (10-18)	14.72 (12-18)

Patients’ follow-ups averaged 28 (14-70) months. Our comparison of hospital stay length between showed a mean length of 13.17 (7-18) days in Group 1 and of 9.75 (5-14) days in Group 2. Length of stay showed a statistically significant difference between groups (p = 0.01). The Karlström-Olerud criteria categorized Group 1 patients’ functional and radiological outcomes as poor in 14 patients, acceptable in five, good in eight, and excellent in one, and as poor in two, acceptable in four, good in 10, and excellent in eight Group 2 patients. We found a statistically significant difference in Karlström-Olerud functional scores between our two groups (p = 0.02) (Table 2).

**Table 2:** Relation between KOOS and length of hospital stay by group.

Characteristic		Group 1	Group 2	p-value
Length of hospital stay (day)		13.17 (7-18)	9.75 (5-14)	0.01
Karlström-Olerud	Poor	14	2	0.01
	Acceptable	5	4	
	Good	8	10	
	Excellent	1	8	

Of the 28 patients in Group 1, nine (with femoral shaft fractures) underwent intramedullary nailing and five (with Gustilo-Anderson type III-b-c fractures), intramedullary nailing following a damage control surgery using external fixation. In total, 12 of 14 patients with fractures involving the articular surface of the distal femur underwent a combination of plate and cannulated screws, whereas two patients with Gustilo-Anderson type III-b-c fractures preferred plate fixation after external fixation. Of the 24 patients with closed femur and tibia fractures, 13 patients with femoral shaft fractures underwent primary fixation with intramedullary nailing and 11, with a combination of plate and cannulated screws. Moreover, 13 patients with tibial shaft fractures underwent intramedullary nailing and 11, a combination of plate and cannulated screws for their fractures involving the articular surface of their proximal tibiae.

In total, two patients with type III-c open fractures underwent vascular repair. Their subsequent insufficient circulation required amputation. Moreover, four patients in the open fracture group underwent dual-plating knee arthrodesis due to the development of osteoarthritis at follow-up. We found that six patients showed femoral fracture nonunion, four of which had open fractures and two, closed ones. Moreover, two patients developed tibia nonunion, one in the open group and the other in the closed group. We diagnosed osteomyelitis in five (17%) patients in Group 1 and in one (4%) patient in Group 2 (Table 3).

**Table 3:** Complications and their distribution.

Complications	Group 1	Group 2
Amputation	2 (7%)	0
Knee arthrodesis	4 (14%)	0
Femoral nonunion	4 (14%)	2 (8%)
Tibial nonunion	1 (3%)	1 (4%)
Osteomyelitis	5 (17%)	1 (4%)
Superficial infection	1 (3%)	1 (4%)

## DISCUSSION

This study functionally compared patients who had floating knee injuries with open femoral and tibial fractures and those who had floating knee injuries with closed femoral and tibial fractures. No study in the literature has compared open and closed fractures.

Previous studies suggest early final fixation of floating knee injuries as advantageous^9^ ^),(^ ^10^ in orthopedic surgeries as it reduces hospital stay length. ^(^ ^11^ Open fractures, however, have been considered disadvantageous in this regard. Although we aimed at early fixation for both patient groups, the transition to internal fixation after infection control with external fixators in the open fracture group prolonged those patients’ hospital stay.

Our comparison of Karlström-Olerud functional outcomes between groups showed better outcomes in the closed fracture group (p = 0.02). Similar studies support the good outcomes of closed fractures. ^(^ ^12^ Kulkarni et al. ^(^ ^13^ found that floating knee injuries suffer the influence of open or closed fractures, segmental nature, additional injuries, and intraarticular surfaces. Our study ignored floating knee injuries with segmental fractures. We found no statistically significant difference in fracture types between groups. This facilitated our evaluation of patients with open and closed fractures, rendering it more objectively and independent of other factors. Chouhan et al. ^(^ ^14^ compared Fraser subtypes considering that fracture types would affect outcomes, showing that IIA fractures had better functional outcomes than IIB and IIC ones. From this point of view (and considering that Fraser subtypes would affect the outcomes), our study compared Fraser subtypes between groups and found no significant difference between them, making our study comparable regarding open-closed fractures.

Floating knee injuries also show complications due to their high-energy nature. Rollo et al. ^(^ ^15^ found compartment syndrome in eight patients, open fractures in 60, and partial amputation in 24, having to perform total amputation on three patients. We amputated two patients in the open floating injury group due to insufficient circulation after vascular repair. Floating knee injuries can seriously damage bones and soft tissues and may even progress to amputation in patients with open fractures.

It would be inaccurate to consider floating knee injuries as isolated bone lesions as these traumas can also injure the soft tissues around and inside the knee. A study investigating concomitant ligamentous and meniscal tissue injuries reported that they co-occurred by meniscus, anterior cruciate ligament, and posterior cruciate ligament injuries, which required treatment after a careful physical examination. ^(^ ^16^ This study ignored additional ligamentous injuries. Further additional and complex traumas in patients may hinder the determination of subgroups in the floating knee classification. ^(^ ^17^

Other system and organ injuries often follow floating knee injuries. Although our study excluded patients with additional injuries, two patients in the open fracture group showed vascular injuries. The literature has reported poor prognostic outcomes for patients with vascular injuries, ^(^ ^18^ agreeing with our results.

Fixation methods also vary in floating knee injuries, provoking discussions on which fracture should be fixed first and by which implant. Dwyer et al. ^(^ ^9^ reported that treating femur fractures by external fixation reduced knee range of motion due to quadriceps muscle dysfunction, but their method for fixating tibial fractures had no effect on outcomes. Our study ignored comparing groups by implant types and fixation methods as they scarcely affect outcomes due to similar fracture types.

Our study diagnosed osteomyelitis in 20% of patients in the open fracture group and in 4% in the closed fracture group. The case series in Chouhan et al. ^(^ ^14^ included 27 patients, finding infections and osteomyelitis in 25% and 11% of them, respectively. Shahzad et al., ^(^ ^19^ on the other hand, found femoral and tibial infections in 16.9% and 20% of their 65 patients, respectively. Our study results and literature data have shown that floating knee injuries increase the risk of osteomyelitis due to its high-energy nature and surgical procedures, a process triggered by the open fracture pattern since open fracture management is closely related to both negatives in the process of fracture union and infections. ^(^ ^20^

Our study has a number of limitations, including its retrospective setting and no examination of the effects of ligamentous injuries on outcomes. Moreover, how fixation methods and length of transition from external to internal fixation affect outcomes remains unknown.

## CONCLUSION

Floating knee injuries involving the femur and tibia configure rare injuries.

In conclusion, floating knee injuries with open femur and tibia fractures show poorer functional outcomes than those with isolated closed fractures.
